# 

**Published:** 1925

**Authors:** 


### The Library of the Bristol Medico-Chirurgical Society.

*The following publications have been received since the publication of the list in October,* 1925.

*December,* 1925.

| | | |
|---|---|---|
| Dr. Serge Voronoff (1) .. .. .. .. | 1 | volume. |
| Medical Officer of Health, Bristol (2) .. .. | 1 | ,, |
| Rockefeller Foundation (3) .. .. .. | 1 | ,, |
| London School of Hygiene and Tropical Medicine (4) | 1 | ,, |

Unbound periodicals have been received from Mr. Hey Groves.

### THE ONE HUNDRED AND FOURTEENTH LIST OF BOOKS.

The figures in round brackets refer to the figures after the names of the donors. The books to which no such figures are attached have either been bought from the Library Fund or received through the *Journal.*

**Andrewes, F. W., and others.** *Diphtheria, its Bacteriology, Pathology, and Immunology* (Medical Research Council) 1923

**Beck, C.**.. .. .. *Crippled Hand and Arm* .. .. .. .. .. 1925

*Collected Addresses and Laboratory Studies,* Vol. I. .. .. .. (4) 1925

**Girdlestone, G. R.** *Diagnosis and Treatment of Tuberculosis of the Hip* .. .. .. .. .. .. .. .. .. .. 1925

**Greenfield, J. G., and Carmichael, E. A.** *Cerebro-Spinal Fluid in Clinical Diagnosis* .. .. .. .. .. .. .. 1925

**Hare, H. A., and Appleman, L. F.** *Progressive Medicine*, Vols. 1—4 1923
*Progressive Medicine*, Vols. 2, 4 .. .. .. 1924
*Progressive Medicine*, Vols. 1—3 .. .. .. 1925
**Henry, G. W.** .. *Essentials of Psychiatry* .. .. .. .. .. 1925
**Kenwood, H. R.** .. *Public Health Laboratory Work (Chemistry)* 8th Ed. 1925
**Kettle, E. H.** .. *Pathology of Tumours* .. .. .. 2nd Ed. 1925
*Methods and Problems of Medical Education* (3rd Series) .. .. (3) 1925
**Miles, A.** .. .. *Guide to the Study of Medicine* .. .. .. 1925
**Morison, R.** .. .. *Abdominal and Pelvic Surgery for Practitioners* 1925
**Morten, Honnor** .. *Nurses' Dictionary* .. .. .. .. 11th Ed. 1925
**Myers, J. A.** .. .. *Vital Capacity of the Lungs* .. .. .. .. 1925
**Norton, Felicie** .. *Coaching Manual for Midwifery Students* .. 1925
**Page, C. M., and Bristow, W. R.** *Treatment of Fractures and Dislocations in General Practice* .. 2nd Ed. 1925
**Paul, F. T.** .. .. *Selected Papers, Surgical and Pathological* .. 1925
**Ross, J. M.** .. .. *Post-mortem Appearances* .. .. .. .. 1925
**Voronoff, Serge** .. *Étude sur la Vieillesse et le Rejeunissement* (1) 1926
**Werner, L.** [Ed.] .. *Swanzy's Handbook of Diseases of the Eye* 13th Ed. 1925

### TRANSACTIONS, REPORTS, JOURNALS, ETC.

British Journal of Surgery, October .. .. .. .. .. .. .. .. 1925
Medical Annual—General Index, 1915—1924 .. .. .. .. .. .. 1925
Guy's Hospital Reports, Vol. 75, No. 4, October .. .. .. .. 1925
St. Thomas's Hospital Reports, Vol. 47 .. .. .. .. .. .. 1925
Annual Report of the Medical Officer of Health, Bristol, 1924 (2) 1924

### Local Medical Notes.

---

EXAMINATION RESULTS.

University of Bristol.—Students of the University have recently passed the following examinations :—

M.B., Ch.B.—*First Examination* (*Pass*) *:* T. Evans, A. M. B. Walker. *Second Examination, Part I.* (*Pass*) *:* J. S. Adamson, A. C. Price, N. L. Price. *In Elementary Anatomy only :* H. F. C. Corfield, R. M. Hickman, D. A. C. Price. *Second Examination* (*Pass*) *:* T. A. Barnabas, B. J. Boulton, E. Jacomb, R. D. Jenkins. *Final Examination, Part I.* (*Pass*), *including Forensic Medicine and Toxicology :* H. M. Aldwinckle, C. France-Hayhurst, F. W. T. Hughes, E. May, R. E. Satchwell, S. P. Taylor. *Final Examination, Part II.* (*Pass*) (*completing examination*) *:* D. H. Beatson, H. W. Brassington, B. F. V. Dawkins, C. H. Durnford, W. S. Ormiston, M. E. J. Packer, Yolande de la Pasture, C. P. Porter, T. W. Ware. *In Group II.* (*completing examination*) *:* E. E. Benson, M. P. Posthuma, K. M. Willmore. *In Group I.* (*completing examination*) *:* C. F. R. Killick. *In Public Health only* (*completing examination*) *:* C. T. Hyatt, H. J. Satchwell.

M.D.—F. J. Hector, M.B., Ch.B., by dissertation on " Some Effects of Diphtheria upon Carbohydrate Metabolism."

L.D.S.—J. Partridge. *First Examination, Pass in Physics* (*completing examination*) *:* E. R. Carpenter, T. A. Satchwell. *In Physics only :* A. N. Moon. *In Chemistry only :* B. J. Shapland. *In Anatomy and Physiology* (*completing examination*)*:* P. H. Slater, E. J. Tanner, G. W. Vowles, P. J. Whiteman, A. E. Wilkins. *In Dental Anatomy only :* A. L. Gilchrist, L. W. G. Jones. *Final Examination* (*Pass*) *:* C. S. Cossham, S. Phillips. *Second Examination* (*Pass*) *:* R. W. Pinniger. *In Dental Mechanics* (*completing examination*) *:* A. M. Thoseby. *In Dental Mechanics and Dental Metallurgy* (*completing examination*) *:* C. S. Wilde. *In Dental Metallurgy and Dental Materia Medica only :* J. H. Edwards, A. L. Gilchrist.

B.D.S.—*Second Examination, Pass in Anatomy, Physiology and Histology* (*completing examination*) *:* W. M. Evans.

D.P.H.—*Pass, in Part II.* (*completing examination*) *:* F. P. Mackie, N. A. M. Rodger.

Conjoint Board.—M.R.C.S., L.R.C.P. (*completing examination*).—M. E. Drew, S. P. Taylor.

L.D.S., R.C.S.—*First Examination, Part I., Pass* (*Mechanical Dentistry*) *:* W. Woolley. *Part II.* (*Dental Metallurgy*) *:* J. F. V. Sellin. *Part III.* (*b*) (*Dental Anatomy*) *:* A. J. Batten, J. F. V. Sellin, R. G. J. Tovey.

Society of Apothecaries of London.—*Pass* (*in Midwifery*) *:* B. Davis, P. H. Knowles, M. E. G. Wilkinson. *In Anatomy :* T. A. Barnabas. *In Surgery :* M. E. G. Wilkinson. *In Forensic Medicine :* W. O. H. Evans, M. E. G. Wilkinson, P. H. Knowles. *In Biology :* G. Smith, I. M. Williams. *In Medicine :* P. H. Knowles, M. E. G. Wilkinson. *In Surgery :* P. H. Knowles, W. O. H. Evans. Diploma of the Society was granted to M. E. G. Wilkinson.

Miss C. I. Ham has been appointed Temporary Demonstrator in Physiology for the Autumn and Spring Terms of the current session.

Dr. S. Hardy Kingston has been appointed as Assistant Lecturer in Public Health.

Wedding.—Salisbury—Wright.—On December 9th, at St. Mary's Church, Broughton, Lincs., Walter Salisbury, M.S., M.D. Lond., F.R.C.S. Eng., younger son of Mr. and Mrs. F. G. Salisbury, of 4 Durdham Park, to Constance Mary, only child of Mr. and Mrs. William Wright, of Broughton Vale.

**Bristol Royal Infirmary. Opening of New Wing of the Nurses' Home.**—On Wednesday, December 9th, 1925, an interesting ceremony took place at the Bristol Royal Infirmary, when the New Wing of the Nurses' Home was opened by Dame Mary Monica Wills, whose late husband, Mr. H. H. Wills, so generously gave a large sum of money to the Royal Infirmary for that purpose. Her Grace The Dowager Duchess of Beaufort, who is the President of the Ladies' Needlework Guild, and has been associated with the Royal Infirmary for many years, had consented to take the Chair, but when the time came was unfortunately unable to do so owing to illness. Her place was taken, almost at a moment's notice, by her daughter-in-law, Her Grace the Duchess of Beaufort, who with the utmost kindness for the purpose put off an engagement in London.

Before proceeding to the New Dining Hall, Her Grace the Duchess of Beaufort called upon Dame Mary Monica Wills to open the doors, whereupon Sir George Oatley handed her a golden key with which to perform the ceremony, and Bishop Clifford entered the building saying: " Peace be upon this House."

On the platform in the New Dining Hall were Her Grace The Duchess of Beaufort, the Lord Mayor and Lady Mayoress, Bishop Clifford, Dame Mary Monica Wills, Colonel P. G. Robinson, D.S.O. (the President), Alderman F. Sheppard (the Chairman), Mr. Kossuth Robinson, Mr. Richardson Cross, Mrs. Harford, Sir George Oatley (the Architect), Miss Baillie, the Rev. H. H. Hawkins (the Chaplain), Mr. Ellis Smith (the Secretary), Miss E. E. P. MacManus (the Matron).

In a thoughtful speech Colonel Robinson reviewed the past, present and future requirements of the Bristol Royal Infirmary, and explained that with each expansion of the number of beds available for patients it was necessary to provide quarters for the Nurses who were to nurse the additional patients. The splendid Dining Hall in which the company were assembled will hold two hundred persons at a sitting. Excellent pantries are provided, and the kitchen department is being entirely remodelled on modern lines with steam, gas and electric kitchen apparatus, including a washing-up machine and a potato peeler, thus completing one of the most up-to-date Nurses' Homes in the West of England.

Medals were presented to Miss Baillie, the late Matron, and to some of the nurses. The whole party made a tour of inspection round the building, returning for tea to the Dining Hall, where the Sisters and Resident Doctors made admirable hosts.

### PUBLICATIONS RECEIVED.

From Baillière, Tindall & Cox, London:

*Selected Papers, Surgical and Pathological.* By F. T. Paul, D.Sc. (Liverp.) (Hon. Caus.), Ch.M. (Liverp.), F.R.C.S. (Eng.). 1925. Price 15s.

*Essentials of Psychiatry.* By George W. Henry, M.D., with a chapter on Psychiatric Nursing. By Adele Poston, R.N. 1925. Price 13s. 9d.

From Messrs. Faber and Gwyer Ltd., London:

*Coaching Manual for Midwifery Students.* By Felicie Norton, S.R.N. 1925. Price 1s. 6d.

*Nurses' Pronouncing Dictionary.* By Honnor Morten. Eleventh Edition. 1925. Price 3s. 6d.

From H. K. Lewis & Co., London:

*Public Health Laboratory Work (Chemistry).* By Henry R. Kenwood, C.M.G., M.B., F.R.S. (Edin.), Eighth Edition. 1925. Price 12s. 6d. net.

*The Pathology of Tumours.* By E. H. Kettle, B.S. (Lond.). Second Edition. 1925. Price 12s. 6d. net.

*Swanzy's Handbook of the Diseases of the Eye, and their Treatment.* Edited by Louis Werner, M.B., F.R.C.S.I., Sen. Mod., Univ. Dub. Thirteenth Edition. 1925. Price 21s. net.

*What to do in Cases of Poisoning.* By W. Murrell, M.D., F.R.C.P. Thirteenth Edition. Revised by P. Hamill, M.D., D.Sc., F.R.C.P. 1925. Price 4s. 6d. net.

*Manipulative Surgery.* By A. G. Timbrell Fisher, M.C., F.R.C.S. 1925. Price 7s. 6d. net.

From Humphrey Milford, Oxford University Press:

*Abdominal and Pelvic Surgery for Practitioners.* By Rutherford Morison, Hon. M.A., and D.C.L., Hon. LL.D., M.B., F.R.C.S. (Edin. and Eng.). 1925. Price 8s. 6d. net.

*Post-mortem Appearances.* By Joan M. Ross, M.B., B.S. (Lond.), M.R.C.S., L.R.C.P. Preface by E. H. Kettle, M.D. 1925. Price 7s. 6d. net.

*Diagnosis and Treatment of Tuberculosis of the Hip.* By G. R. Girdlestone, B.M. F.R.C.S. 1925. Price 8s. 6d. net.

From Messrs. Oliver & Boyd, London:

*A Guide to the Study of Medicine.* By Alexander Miles, M.D., F.R.C.S., LL.D. 1925. Price 3s. net.

From Messrs. Wakley & Son Ltd., London:

*Guy's Hospital Reports*, Vol. 75, No. 4, October, 1925. Annual Subscription, £2 2s. 0d. net or 12s. 6d. net per issue.

From Messrs. J. Wright & Sons Ltd., Bristol:

*British Journal of Surgery,* October, 1925. Price 12s. 6d. net. Annual Subscription, 42s. net.

*Medical Annual General Index.* 1912—1924. 1925. Price 12s. 6d. net.

### INDEX.

Adams, A. W.—A Comparative Review of Spinal and Peripheral Nerve Injuries, 32.
Anæsthetics, The Comparative Efficiency of Local.—E. Watson-Williams, 164.
Appendicitis as a Cause of Intestinal Obstruction.—R. Warren, 23.
Appointments, 72.
Asthma, A Note on.—J. R. Charles, 84

Books, Reviews of—
Abdominal and Pelvic Surgery for Practitioners.—Rutherford Morison, 234.
Acute Infectious Diseases.—J. D. Rolleston, 131.
Alcohol in Medical Practice.—Courtenay C. Weeks, 233.
Allbutt, Sir T. C.—Arteriosclerosis, 232.
Allbutt, Sir T. C.—Preface to Post-Graduate Lectures, 136.
Anæmia : its Causes and Modern Trestment.—A. W. Fuller, 45.
Annals of the Pickett-Thomson Research Laboratory, 237.
Arteriosclerosis.—Sir T. Clifford Allbutt, 232.
Artificial Sunlight and its Therapeutic Uses. — Francis Howard Humphris, 236.
Bacteriology of Food, The.—C. Dukes, 129.
Barton, E. A.—Essentials of Infant Feeding, 186.
Beattie, J. Martin and Dickson, W. E. Carnegie.—A Text-book of Pathology, General and Special, 239.
Beck, Carl.—The Crippled Hand and Arm, 234.
Berkeley, Comyns.—Gynæcology for Nurses and Gynæcological Nursing, 238.
Bland-Sutton, Sir J.—Orations and Addresses, 127.
Brand, A. T., and Keith, J. R.—Clinical Memoranda, 47.
Breast, On the.—D. C. L. Fitz-William, 134.
Cancer : Postgraduate Lectures.—H. L. Paterson (ed.), 183.
Cancer Surgery, Some Encouragments in. — G. Grey Turner, 182.

Books, Reviews of (continued)—
Canned Foods in Relation to Health.—William G. Savage, 238.
Carless, A.—Manual of Surgery, 182.
Carson, H. W.—Modern Operative Surgery, 133.
Chemical and Physiological Properties of the Internal Secretions.—E. C. Dodds and F. Dickens, 232.
Chronic Intestinal Stasis.—A. C. Jordan, 131.
Clayton Green, W. H. (ed.).—Pye's Surgical Handicraft, 136.
Clinical Memoranda.—A. T. Brand and J. R. Keith, 47.
Clinical and Operative Gynæcology.—J. M. Munro Kerr, 51.
Clinical Researches in Acute Abdominal Disease.—Z. Cope, 184.
C. M. B. Examination Questions and Model Answers, 238.
Consumption, The Modern Nursing of.—Jane H. Walker, 176.
Coombs, Carey F.—Rheumatic Heart Disease, 43.
Cope, Z.—Clinical Researches in Acute Abdominal Disease, 184.
Crippled Hand and Arm, The.—Carl Beck, 234.
Crofton, W. M.—An Outline of Endocrinology, 185.
Culpin, M.—The Nervous Patient, 46.
De Monte.—Primary Problems of Medical Psychology, 186.
Dermatology, An Introduction to.—Sir N. Walker, 130.
Diagnosis and Treatment of the Infectious Diseases.—F. H. Thomson, 45.
Dodds, E. C., and Dickens, F.—Chemical and Physiological Problems of the Internal Secretions, 232.
Dosage Tables for Deep-Therapy.—F. Voltz, 137.
Dukes, C.—The Bacteriology of Food, 129.
Dyspepsia, Lectures on.—R. Hutchison, 181.

BOOKS, REVIEWS OF (continued)—
Ellis, H. A.—An Explanation of Hydrogen Ion Concentration, 187.
Endocrinology, An Outline of.—W. M. Crofton, 185.
Epidemic Encephalitis.—A. J. Hall, 131.
Essentials of Infant Feeding.—E. A. Barton, 186.
Extra Pharmacopœia of Martindale and Westcott, 48.
Facial Surgery.—H. P. Pickerill, 135.
Fairburn, J. S.—Gynæcology with Obstetrics, 174.
Fifield, L. R.—Minor Surgery, 181.
Fisher, A. G. T.—Internal Derangement of the Knee Joint, 47.
FitzWilliam, D. C. L.—On the Breast, 134.
Flexner, A.—Medical Education, 177.
Fuller, A. W.—Anæmia, 45.
Gardiner, F.—Handbook of Skin Diseases, 52.
Goitre.—F. de Quervain, 135.
Groves, E. W. Hey.—Surgical Operations, 174.
Groves, Ernest W. Hey.—A Synopsis of Surgery, 235.
Groves, E. W. Hey.—A Text-book for Nurses, 237.
Gynæcology for Nurses and Gynæcological Nursing.—Comyns Berkeley, 238.
Gynæcology with Obstetrics.—J. S. Fairburn, 174.
Haig, H. A. — Histology of Tumours, 49.
Hall, A. J. — Epidemic Encephalitis, 131.
Hall, P.—Ultra-Violet Rays, 137.
Ham, B. Burnett.—Handbook of Sanitary Law, 52.
Handbook of Skin Diseases.—F. Gardiner, 52.
Health and Environment.—L. Hill, 180.
Hill, L.—Health and Environment, 180.
Humphris, Francis Howard.—Artificial Sunlight and its Therapeutic Uses, 236.
Hutchison, R. — Lectures on Dyspepsia, 181.
Hydrogen Ion Concentration.—H. A. Ellis, 187.

BOOKS, REVIEWS OF (continued)—
Jordan, A. C.—Chronic Intestinal Stasis, 131.
*Journal of Cancer, The*, 50.
Kennedy, A. M.—Parasitology, 185.
Kerr, J. M. Munro.—Clinical and Operative Gynæcology, 51.
Kettle, E. H.—The Pathology of Tumours, 240.
Kingscote, E. — Movement in Organic Disease, 46.
Knee Joint, Internal Derangement of.—A. G. T. Fisher, 47.
Laird, J.—Tuberculosis, 132.
Landmarks and Surface Markings of the Human Body.—L. Bathe Rawling, 48.
Leprosy.—Sir L. Rogers, 127.
Leslie, R. Murray.—Pneumonia, 44.
McBride, P.—Psycho - Analysis Analysed, 175.
McDonagh, J. E. R.—The Nature of Disease, 172.
Manson's Tropical Diseases.—P. H. Manson-Bahr, 178.
Marshall, C. Jennings and Piney, A. — Text - book of Surgical Pathology, 240.
Martindale, W. H., and Westcott, W. W.—The Extra Pharmacopœia, 48.
May, C. H., and Worth, C.—A Manual of Diseases of the Eye.—50.
Medical Education.—A. Flexner, 177.
Medical Psychology. — Ch. de Montet, 186.
Middle Age and Old Age.—L. Williams, 176.
Minor Surgery.—L. R. Fifield, 181.
Morison, Rutherford.—Abdominal and Pelvic Surgery for Practitioners, 234.
Muir, J.—Practical X-ray Work, 131.
Muir, R.—Text-book of Pathology, 49.
Myers, J. A.—Vital Capacity of the Lungs, 234.
Nature of Disease.—J. E. R. McDonagh, 172.
Nervous Patient, The. — M. Culpin, 46.
Orations and Addresses.—Sir J. Bland-Sutton, 127.

Books, Reviews of (continued)—
Orthopædic Surgery, The Advance of.—A. H. Tubby, 137.
Parasitology.—A. M. Kennedy, 185.
Pathology, Text - book of. — R. Muir, 49.
Pathology of Tumours, The.— E. H. Kettle, 240.
Pernicious Anæmia and Aplastic Anæmia.—A. Sheard, 132.
Pickerill, H. P.—Facial Surgery, 135.
Pneumonia.—R. Murray Leslie, 44.
Postgraduate Lectures, 136.
Practical Chiropody.—E. G. V. Runting, 238.
Practical Medicine Series, Vol. I., 132.
Psycho-Analysis Analysed. — P. McBride, 175.
Pye's Surgical Handicraft, 136.
Quervain, F. de.—Goitre, 135.
Rawling, L. Bathe.—Landmarks and Surface Markings, 48.
Rea, R. Lindsay.—A Preliminary Report on the Treatment of Interstitial Keratitis, 236.
Report on the Treatment of Interstitial Keratitis, A Preliminary.—R. Lindsay Rea, 236.
Rheumatic Heart Disease.— Carey F. Coombs, 43.
Rogers, Sir L.—Leprosy, 127.
Rolleston, J. D.—Acute Infectious Diseases, 131.
Runting, E. G. V.—Practical Chiropody, 238.
St. Andrew's Institute, Reports of the, Vol. II., 175.
Savage, William G.—Canned Foods in Relation to Health, 238.
Saywell, Evelyn. — Side - lights from the New Psychology, 186.
Schmidt, P.—Steinach Operation, 183.
Sheard, A.—Pernicious Anæmia and Aplastic Anæmia, 132.
Sidelights from the New Psychology.—E. Saywell, 186.
Steinach Operation.—P. Schmidt, 183.
Surgery, Manual of.—A. Carless, 182.
Surgical Operations.—E. W. Hey Groves, 174.

Books, Reviews of (continued)—
Synopsis of Medicine.— H. Letheby Tidy, 235.
Synopsis of Special Subjects, 184.
Synopsis of Surgery.—Ernest W. Hey Groves, 235.
Text-book for Nurses.—E. W. Hey Groves, 237.
Text-book of Pathology, General and Special.—J. Martin Beattie and W. E. C. Dickson.—239.
Text-book of Surgical Pathology. —C. Jennings Marshall and A. Piney, 240.
Thomson, H. — Infectious Diseases, 45.
Tidy, H. Letheby.—Synopsis of Medicine, 235.
Tubby, A. H.—Advance of Orthopædic Surgery, 137.
Tuberculosis.—J. Laird, 132.
Tumours, Histology of.—H. A. Haig, 49.
Turner, G. Gray. — Cancer Surgery, 182.
Ultra-Violet Rays.—P. Hall, 137
Vital Capacity of the Lungs.— J. A. Myers, 234.
Voltz, F.—Deep Therapy Dosage Tables, 137.
Walker, Jane H.—Nursing of Consumption, 176.
Walker, Sir N.—Dermatology, 130.
Weeks, Courtenay C.—Alcohol in Medical Practice, 233.
Williams, L.—Middle Age and Old Age, 176.
X-ray Work.—J. Muir, 131.

*Bristol Medico-Chirurgical Journal* for 1926, 241.
Bristol Medical School Dinner, 241.
Bristol Medico-Chirurgical Society, 55, 142.
British Medical Association.— Annual Meeting, 190.

Carleton, H. H.—Some Problems in Pulmonary Disease, 211.
Carwardine, T.—Continuity and Change, 201.
Charles, J. R.—A Note on Asthma, 84.
Clarke, C.—Obituary, 66.
Colston Research Society, 141.
Continuity and Change.—T. Carwardine, 201.
Crematorium for Bristol, 55.

Discussions :
Appendicitis as a Cause of Intestinal Obstruction.—R. Warren, 23. Discussed by A. R. Short, 29 ; F. C. Walters, 30 ; C. Chitty, 30 ; D. G. C. Tasker, 31 ; F. Lacy Firth, 31.
Carcinoma of the Œsophagus (including the Deep Pharynx). —A. J. Wright, 58. Discussed by J. P. I. Harty, 59 ; C. E. K. Herapath, 59 ; C. F. Walters, 59 ; F. Lacy Firth, 59 ; E. Watson - Williams, 59 ; C. Corfield, 59.
Diagnosis of the Dyspepsias.— A. R. Short, 88. Discussed by C. A. Moore, 100 ; D. A. Alexander, 100 ; A. W. Adams, 100.
Encephalitis Lethargica [Opening of Discussion by F. H. Edgeworth], 60. Discussed by D. S. Davies, 60 ; H. H. Carleton, 60 ; R. A. Askins, 61 ; E. R. Chambers, 61 ; J. Wallace, 61 ; A. E. J. Lister, 61 ; R. G. Gordon, 61.
Problems in Pulmonary Disease. —H. H. Carleton, 211. Discussed by F. G. Bergin, 221 F. J. A. Mayes, 222 ; F. H. Edgeworth, 222 ; J. O. Symes, 222 ; C. F. Walters, 222 ; J. A. Nixon, 222 ; Carey F. Coombs, 223.

Dunbar, Eliza W.—Obituary, 197.
Dyspepsias, Diagnosis of the.— A. R. Short, 88.

Editorial Committee, Changes in, 54.
Evans, H. L.—Obituary, 151.
Examination Results, 71, 155, 246.

Focal Infection in the Nasal Sinuses. — P. Watson - Williams, 121.

Gordon, W.—Posture in Physical Examination of the Heart, 224.
Griffiths, L. M.—Obituary, 61.

Heart, Physical Examination of the.—W. Gordon, 224.

Library of the Bristol Medico-Chirurgical Society, 69, 152, 198, 244.
Local Medical Notes, 71, 154, 246.

Medical Faculty of the University of Bristol, The, 138.

New Buildings of the University of Bristol.—Notes on, 139 ; Royal Opening of, 73.
Norgate, R. H.—Obituary, 65.

Obituary.—C. Clarke, 66 ; Eliza W. Dunbar, 197 ; H. L. Evans, 151 ; L. M. Griffiths, 61 ; R. H. Norgate, 65 ; A. B. Prowse, 149 ; W. C. Swayne, 192 ; J. Taylor, 145.
Open-Air Hospital School, 53.

Postgraduate Medical Education. —J. O. Symes, 1.
Presidential Address. — T. Carwardine, 201 ; J. O. Symes, 1.
Problems in Pulmonary Disease.— H. H. Carleton, 211.
Prowse, A. B.—Obituary, 149.

Royal Visit, The, 138, 198.

Sargent, P.—Surgery of the Nervous System, 101.
Scarlet Fever, The Diagnosis of.— J. Wallace, 157.
Short, A. R.—Diagnosis of the Dyspepsias, 88.
Spinal and Peripheral Nerve Injuries.—A. W. Adams, 32.
Surgery of the Nervous System.— P. Sargent, 101.
Swayne, W. C.—Obituary, 192.

Taylor, J.—Obituary, 145.

University of Bristol, The, 191.
The Medical Faculty of, 138.
Notes on the New Buildings of, 139.
Royal Opening of the New Buildings, 73.
University College of the South-West, The, 188.

Wallace, J.—The Diagnosis of Scarlet Fever, 157.
Warren, R.—Appendicitis as a Cause of Intestinal Obstruction, 23.
Watson-Williams, E.—The Comparative Efficiency of Local Anæsthetics, 164.
Watson-Williams, P.—Diagnosis of Focal Infection in the Nasal Sinuses, 121.

